# Walrasian Equilibrium-Based Incentive Scheme for Mobile Crowdsourcing Fingerprint Localization

**DOI:** 10.3390/s19122693

**Published:** 2019-06-14

**Authors:** Tao Yu, Linqing Gui, Tianxin Yu, Jilong Wang

**Affiliations:** 1 Institute of Network Science and Cyberspace, Tsinghua University, Beijing 100084, China; yutao@cutech.edu.cn (T.Y.); wjl@tsinghua.edu.cn (J.W.); 2 Department of Electronic and Optical Engineering, Nanjing University of Science and Technology, Nanjing 210094, China; yutianxin@njust.edu.cn

**Keywords:** localization, fingerprinting, crowdsourcing, equilibrium

## Abstract

Mobile crowdsourcing has been exploited to collect enough fingerprints for fingerprinting-based localization. Since the construction of a fingerprint database is time consuming, mobile users should be well motivated to participate in fingerprint collection task. To this end, a Walrasian equilibrium-based incentive mechanism is proposed in this paper to motivate mobile users. The proposed mechanism can eliminate the monopoly of the crowdsourcer, balance the supply and demand of fingerprint data, and maximize the benefit of all participators. In order to reach the Walrasian equilibrium, firstly, the social welfare maximization problem is constructed. To solve the original optimization problem, a dual decomposition method is employed. The maximization of social welfare is decomposed into the triple benefit optimization among the crowdsourcer, mobile users, and the whole system. Accordingly, a distributed iterative algorithm is designed. Through the simulation, the performance of the proposed incentive scheme is verified and analyzed. Simulation results demonstrated that the proposed iterative algorithm satisfies the convergence and optimality. Moreover, the self-reconstruction ability of the proposed incentive scheme was also verified, indicating that the system has strong robustness and scalability.

## 1. Introduction

Recently, driven by the growing demand for indoor location-based services (LBS), indoor localization has attracted a lot of research attention [1]. Since it is difficult for indoor mobile devices to receive GPS signals, alternative techniques such as Ultra Wideband (UWB), Radio Frequency Identification (RFID) and ZigBee have been developed for indoor localization. Among these techniques, Wi-Fi becomes more and more popular because Wi-Fi infrastructures have been widely deployed in indoor environments [2]. In the literature of Wi-Fi indoor localization, plenty of approaches adopt fingerprinting as a metric for location determination [3,4,5]. Here fingerprinting denotes location-related measurement, e.g., received signal strength (RSS) or channel state information (CSI). Compared to RSS, CSI fingerprint has much larger spatial resolution [6]. Moreover, CSI fingerprint is more robust than RSS [7]. As a result, a CSI-based localization scheme usually has better accuracy than an RSS-based scheme. Therefore, this paper focuses on indoor localization based on CSI fingerprint information.

During the offline stage of fingerprinting-based localization, the rich set of embedded sensors in mobile devices can be exploited to obtain the traces and positions of mobile users. The embedded sensors include camera, gyroscope, GPS, accelerator, light sensor, and digital compass [8]. For example, in [9], gyroscope and accelerator are leveraged to estimate the real-time position of each mobile user. The position and a pilot signal are then put into a packet which will be reported by a mobile user to a nearby wireless access point (AP). The pilot signal is utilized for the estimation of CSI. Upon receiving the packet, the AP can not only acquire the position of the mobile user, but also the CSI between itself and the mobile user. Thus a fingerprint is constructed, composed of the position of the mobile user and the CSI between the mobile user and the AP.

One technical difficulty of fingerprinting-based localization is that the construction of the fingerprint database requires a lot of manpower and material resources, especially when the collection of fingerprints is executed in a large building [10]. In order to solve this problem, mobile crowdsourcing has been exploited for indoor fingerprint positioning [11]. Recently, various crowdsourcing-based indoor localization methods have been proposed [8,12,13]. However, a major challenge for crowdsourcing-based indoor fingerprint localization is how to motivate mobile users to participate in CSI fingerprint collection tasks. The accuracy of fingerprinting-based localization depends on the number of CSI fingerprints collected by mobile devices. If there are not enough mobile user participators, the collected fingerprints will be so limited that satisfactory localization accuracy can hardly be achieved [14]. So the incentive mechanism is very important to motivate mobile users to participate in mobile crowdsourcing.

The incentive mechanism arouses the interests of mobile users to participate in the fingerprint collection task by leveraging appropriate motivation measures [15,16,17,18]. Existing studies showed partiality to the interest of the crowdsourcer and designed incentive mechanisms from the view of the crowdsourcer [19,20,21,22]. Accordingly, the crowdsourcer builds the crowdsourcing platform and designs the incentive mechanism to encourage the participation of mobile uses. As a result, the crowdsourcer can maximize its utility while mobile users will behave truthfully. In this kind of platform-centric mobile crowdsourcing systems, the crowdsourcer dominates the whole incentive design process and tries its best to minimize its own cost. The interests of mobile users are accommodated, but not maximized. Only the rationality of mobile users can be guaranteed so that mobile users earn more than their costs. As a result, the rewards mobile users gain are usually much lower than the real contribution that they have devoted. In order to eliminate this unfairness and care for the interests of all participators, there is an urgent need to design new incentive mechanisms for crowdsourcing-based indoor localizations.

Different from traditional incentive mechanisms, Walrasian equilibrium has an extraordinary advantage that it can simultaneously optimize the benefits for all participators in the task [23,24]. Based on exchange market theory, the optimal state of potential buyers and sellers can be characterized by Walrasian equilibrium [25,26]. At a Walrasian equilibrium, the price and the allocation should satisfy the following three conditions simultaneously: (1) the market clears, (2) both the buyers and sellers can maximize their utilities, and (3) the whole system reaches a Pareto optimal operating point [27]. So Walrasian equilibrium has a remarkable feature that transcends the well-known Nash equilibrium [28]. As a result, this paper focuses on a Walrasian equilibrium-based incentive mechanism for fingerprint data collection crowdsourcing task. In the crowdsourcing-based indoor localization system, the crowdsourcer and mobile users are peers, thus the whole market is open, free, and fair [29]. Walrasian Equilibrium can serve this system well and benefit all participators in a balanced manner [30]. The main contributions of this paper are introduced as follows:

1. In order to well-motivate mobile users to participate in the crowdsourcing fingerprint collection task, this paper proposes an incentive mechanism based on Walrasian equilibrium. The proposed mechanism can eliminate the monopoly of the crowdsourcer, balance the fingerprint supply and demand, and maximize the benefit of all participators, including system social welfare.
2. In order to reach the Walrasian equilibrium, a social welfare maximization problem is constructed and solved. To solve the original optimization problem, a dual-decomposition method is employed, decomposing the maximization of social welfare into the triple benefit optimization among the crowdsourcer, mobile users, and the whole system. Accordingly, a distributed iterative algorithm is designed.
3. Through the simulation, the performance of the proposed incentive scheme is verified and analyzed. Firstly, it is verified that the proposed distributed iterative algorithm satisfies the convergence and optimality. Then the self-reconstruction ability of the proposed excitation scheme is analyzed, which shows that the system has strong robustness and scalability.

The rest of this paper is organized as follows. Section 2 illustrates our system model. Section 3 analyzes the utility functions of all participators. Section 4 presents the formulation and solution of optimization problems. Simulation results and analysis are shown in Section 5. Finally the conclusion is given in Section 6.

## 2. System Model

**Figure 1:**
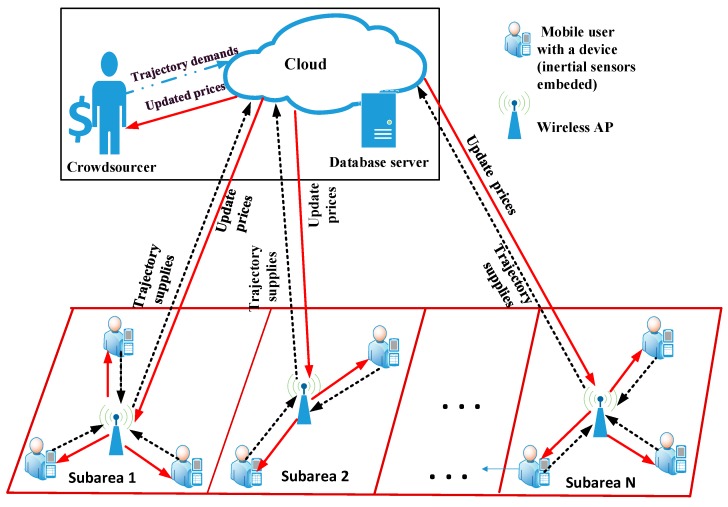
Mobile crowdsourcing model for indoor fingerprint localization.

In the crowdsourcing-based indoor fingerprint localization system, as shown in Figure 1, there is a crowdsourcer, a large group of mobile users, multiple APs, and a cloud which connects the crowdsourcer and the mobile users. The crowdsourcer represents the organization, which demands a number of position fingerprints, e.g., an exhibition center that requires the positions of exhibitors and visitors, or a museum that needs to localize the tourists. The demand of fingerprints by the crowdsourcer will be met by those mobile users which act as indoor fingerprint data providers. In return the mobile users will be rewarded by the crowdsourcer.

The mobile users are distributed inside a building, which is further divided into *N* indoor subareas with its *j*th subarea denoted by *A_j_*. Mobile users are supposed to work in only one subarea. Inside each subarea there is an AP which not only connects the mobile users and the cloud, but also collects the CSI fingerprints from mobile users. Let Λ denote a collection of subareas, i.e., $\Lambda =\left\{A_{1},A_{2},\ldots ,A_{N}\right\}$. Since the subareas may have different sizes, the crowdsourcer requires different number of fingerprints for each subarea. Larger area usually demands more fingerprints. 

When mobile users carry out routine activities in indoor environment, CSI fingerprints of mobile users can be collected by the AP. One fingerprint consists of a temporal position of a mobile user and a sampled CSI reading. The position of the mobile user can be estimated through several localization methods, e.g., localization based on inertial sensors [31] or reference nodes [32]. Walking inside the subarea, the mobile user from time to time reports its position and a pilot signal to the neighboring AP. Based on the pilot signal, the AP measures the CSI of the channel between itself and the mobile user. Combining the position and the CSI, a fingerprint is formed and then reported by the AP to the cloud.

Participating in the fingerprint collection task, mobile users not only consume the energy and the resources of their mobile devices, but also spend their time and sacrifice their privacy. In order to well-incentivize the mobile users, it is strongly suggested to provide them with monetary rewards. Nevertheless, the mobile users should not be rewarded equally because they often contribute differently for the task, i.e., the number of fingerprints they collected are different between each other. In fact, instead of the number of collected fingerprints, another equivalent, but more intuitive metric, namely trajectory distance (or moving distance) of a mobile user, is usually adopted to measure the contribution of the mobile user.

Reduplicative trajectory should be eliminated during the calculation of trajectory distance. Deliberately or not, a mobile user may walk back and forth, causing reduplicative trajectory and fingerprints. When the crowdsourcer computes the reward for one mobile user, the reduplicative trajectory should not be counted. Therefore, the fingerprints at the same positions should be aggregated so as to obtain the effective trajectory distance of a mobile user.

In order to achieve a balance between the benefit of the crowdsourcer and that of mobile users, a Walrasian equilibrium-based incentive mechanism is adopted in our mobile crowdsourcing system. To reach the equilibrium, the crowdsourcer and mobile users need to communicate via the cloud so that they can bargain with each other. The bargaining procedure is briefly introduced as follows. Firstly, the cloud informs the crowdsourcer and mobile users about the initial prices for all subareas. Based on the prices, the crowdsourcer and mobile users compute the trajectory demand and supply, respectively, then report the demand and supply to the cloud. Subsequently, the cloud computes the mismatch between the supplies of mobile users and the demand of the crowdsourcer, updates the prices, and informs both parties about the prices. Once receiving the updated prices, the crowdsourcer and mobile users update the demand and the supplies again, respectively, in the aim of maximizing their own benefits. The updated demand and supplies are reported to the cloud, which then updates the prices again. After several iterations, the equilibrium can be achieved and the optimal prices can be obtained.

## 3. Design of Utility Functions

The crowdsourcer and mobile users both aim to maximize their own profits during the pricing process. In the fingerprint collection task, the prices in different subareas are set to be different because some subareas may be more important than others. For example, in a museum, some halls exhibit rarer collections than other halls. In one subarea, the price is the same for all mobile users therein. Then in subarea *A_j_*, the price for single fingerprint collection is denoted as *P_j_*. Given vector $p={\left(p_{j}\right)}_{A_{j}\in \Lambda }$, which includes the prices of all subareas, the crowdsourcer and mobile users can calculate the trajectory distance they purchase or contribute based on the maximization of their utilities. When the pricing is finished, the demands of the crowdsourcer and the contribution of mobile users are balanced, resulting in optimal social welfare. The design of the utilities and social welfare will be illustrated in the following.

### 3.1. Utility of the Crowdsourcer

The crowdsourcer aims to collect the most fingerprints in each subarea with the least money. In other words, in order to maximize the benefit, the crowdsourcer tries to lower the price while purchase as much trajectory data as possible. Thus, the utility function of the crowdsourcer can be represented by the difference between its payoff and cost, which is formulated as follows:

(1) $$W=\Phi \left(D\right)-\overset{N}{\underset{j=1}{\sum }}p_{j}D_{j},$$

where $D_{j}$ denotes the total trajectory distance that the crowdsourcer demands in subarea $A_{j}$, $D={\left(D_{j}\right)}_{A_{j}\in \Lambda }$ denotes the vector of trajectory distance in all subareas, $p_{j}$ is the single fingerprint price in $A_{j}$, $\sum_{j=1}^{N}p_{j}D_{j}$ is the total remuneration paid by the crowdsourcer to all mobile users, and Φ(⋅) represents the payoff function of the crowdsourcer.

The payoff of the crowdsourcer can be obtained by offering location-based services. In this paper, the localization services were produced from fingerprint data provided by mobile users. Thus. the trajectory distance of mobile users can be regarded as a production factor for developing the economic benefits of the crowdsourcer. As in [30], Cobb–Douglas production function is adopted as the payoff function, explicitly:

(2) $$\Phi \left(D\right)=\sigma \prod_{j=1}^{N}D_{j}^{w_{j}},$$

where σ is a payoff coefficient, $w_{j}$ is an elasticity coefficient for the demand $D_{j}$, and $\sum_{j=1}^{N}w_{j}<1$ is satisfied to preserve the concavity of Φ(D). Therefore, (1) can be equivalently written as:

(3) $$W=\sigma \prod_{j=1}^{N}D_{j}^{w_{j}}-\sum_{j=1}^{N}p_{j}D_{j}.$$

Since the crowdsourcer has to collect enough fingerprint data in each interested subarea to guarantee the overall performance, $D_{j}$ should have a lower bound ${\underline{D}}_{j}$, i.e., $D_{j}\geq {\underline{D}}_{j}$. Therefore, the crowdsourcer can establish the following optimization problems:

(4) $$\max\;\sigma \prod_{j=1}^{N}D_{j}^{w_{j}}-\sum_{j=1}^{N}p_{j}D_{j},$$

$\mathrm{subject}\;\mathrm{to}\;D_{j}\geq {\underline{D}}_{j}$.

Generally, given the price vector $p={\left(p_{j}\right)}_{A_{j}\in \Lambda }$, there exists a vector $D={\left(D_{j}\right)}_{A_{j}\in \Lambda }$, which maximizes the utility function of the crowdsourcer. Here, $D={\left(D_{j}\right)}_{A_{j}\in \Lambda }$ can be regarded as a purchase strategy.

### 3.2. Utility of Mobile User

As to a mobile user $u_{k}$ in $A_{j}$, its effective trajectory distance is denoted as $d_{k}$, which also indicates the fingerprint supplied by $u_{k}$. Then the monetary reward gained by $u_{k}$ is $p_{j}*d_{k}$. To obtain the reward, the mobile user has to devote its time and device resources to fingerprint collection. This devotion generates costs including labor work, the consumption of power, and computation resources of mobile device. The costs vary from person to person because different mobile users can have different availability in time and device resources.

Each mobile user aims to gain the highest reward at the expense of the smallest cost. In other words, in order to maximize the benefit, mobile users wish to have the highest price while supplying the trajectory distance as short as possible. Thus, the utility function $V_{k}$ of user $u_{k}\left(u_{k}\in A_{j}\right)$ can be formulated as:

(5) $$V_{k}=p_{j}d_{k}-\left(a_{k}d_{k}^{2}+b_{k}d_{k}\right),$$

where the cost coefficients $a_{k}$ and $b_{k}$ measure the availability of user $u_{k}$ because difference mobile users can have different level of time and resource availability, $a_{k}>0$ and $b_{k}>0$.

Since each mobile user has limited time and device resource budget to perform the fingerprint collection task, its contributed trajectory distance $d_{k}$ should have an upper bound ${\bar{d}}_{k}$, i.e., $d_{k}\leq {\bar{d}}_{k}$. Therefore, mobile users can create the following optimization problem:

(6) $$max\;p_{j}d_{k}-\left(a_{k}d_{k}^{2}+b_{k}d_{k}\right)$$

$\mathrm{subject}\;\mathrm{to}\;0\leq d_{k}\leq {\bar{d}}_{k}$.

From Equation (6), it can be found that when the prices are determined, each mobile user decides the trajectory distance based on the maximization of its utility function.

### 3.3. Social Welfare

Social welfare refers to the utility of the entire system. So the social welfare can be calculated as the sum utility of the crowdsourcer and mobile users. Thus, we have:

(7) $$J=\left(W+\sum_{k=1}^{M}V_{k}\right),$$

where J represents social welfare, *W* is the utility of the crowdsourcer, and $V_{k}$ denotes the utility of each mobile user. As long as the incentives are real, the monetary rewards paid by the crowdsourcer will become the rewards of all mobile users. So the compensation item in Equation (7) will be eliminated. Then, Equation (7) can be converted to:

(8) $$J=\sigma \prod_{j=1}^{N}D_{j}^{w_{j}}-\sum_{k=1}^{M}(a_{k}d_{k}^{2}+b_{k}d_{k}).$$

From the above formula, it can be found that social welfare depends only on the payoff of the crowdsourcer and the cost of mobile users. The rewards are counteracted in social welfare. Since the price factor is eliminated, the goal of social welfare is to maximize the efficiency of the entire system, i.e., to achieve more benefits at a lower cost. Therefore, social welfare can serve as an important factor to measure the quality of the whole system. Systems with high social welfare tend to be better than systems with low social welfare. As the overall utility increases, it is possible to increase the utility of all individuals.

## 4. Problem Formulation and Algorithm Design

As described in last section, both the crowdsourcer and mobile users want to maximize their own utilities. In brief, the crowdsourcer tries to force prices down so that they could purchase more fingerprint data. However, mobile users want to force prices up so that they can contribute little but gain more monetary rewards. The interest of the crowdsourcer conflicts with that of mobile users. Under specific prices, if the crowdsourcer and mobile users rationally determine their demand and supply by solving the optimization problem in Equations (4) and (6), respectively, there must be a mismatch between the supply of mobile users and the demand of the crowdsourcer. Therefore, the two optimization problems should be solved in a way that can reach an equilibrium with the balance of demands and supplies. To achieve the balance, a negotiation process between the crowdsourcer and mobile users is necessary.

In our crowdsourcing-based fingerprint localization system, the crowdsourcer and mobile users are peers. No one can dominate the fingerprint collection process, so the whole market is open, free, and fair. As a result, the prices of fingerprint collection only depend on the supply (measured by trajectory distance of mobile users) and demand (trajectory distance request from the crowdsourcer) in the market. Based on exchange market theory, an optimal state for both the crowdsourcer and mobile users can be characterized by the Walrasian equilibrium [29].

Next, we first introduce the definition of Walrasian equilibrium and explain its benefit for the crowdsourcing-based fingerprint collection task. Then, the search for Walrasian equilibrium is modeled as social welfare maximization problem with the constraint that supplies and demands match with each other. The global optimization problem is further decoupled into local optimization problems with respect to the crowdsourcer and mobile users. Finally, a corresponding algorithm is proposed to illustrate the whole procedure for reaching Walrasian equilibrium.

### 4.1. Walrasian Euilibrium

Walrasian equilibrium is usually adopted in the market economy to analyze the equilibrium from the view of the whole system. At a Walrasian equilibrium, the following three missions are accomplished simultaneously: first, the market clears, achieving a balance between demands and supplies; second, both buyers and sellers can maximize their payoffs under certain constraints; and third, the overall system reaches a Pareto optimal operating point. In short, a Walrasian equilibrium represents a state beneficial to all participating parties, so it goes beyond the well-known Nash equilibrium.

Based on the aforementioned three features, the definition of Walrasian equilibrium for the crowdsourcing-based fingerprint collection task is introduced as follows.

**Definition** **1.** Walrasian equilibrium in the system means that under the condition of a given price vector $p^{*}={\left(p_{j}^{*}\right)}_{A_{j}\in \Lambda }$, the trajectory distance demand $D^{*}={\left(D_{j}^{*}\right)}_{A_{j}\in \Lambda }$, and trajectory distance supply $d^{*}={\left(d_{k}^{*}\right)}_{u_{k}\in U}$ should meet the following three conditions:

*(1)*  for the crowdsourcer, $D^{*}={\left(D_{j}^{*}\right)}_{A_{j}\in \Lambda }$ is the optimal solution to problem Equation (4);
*(2)*  for any mobile user, $d^{*}={\left(d_{k}^{*}\right)}_{u_{k}\in U}$ is the optimal solution to problem Equation (6);
*(3)*  the optimal demand of the crowdsourcer in each subarea equals to the overall supply of mobile users in each subarea, i.e., $D_{j}^{*}=\sum_{u_{k}\in A_{j}}d_{k}^{*}$, resulting in a clear market.

According to the definition above, both the crowdsourcer and mobile users obtain their optimal utilities, so their satisfaction is simultaneously guaranteed at the Walrasian equilibrium. In addition, the fingerprints supplied by mobile users are fully utilized. Next, we will present how to compute the Walrasian equilibrium and obtain the optimal $\left(p^{*},D^{*},d^{*}\right)$.

### 4.2. Problem Formulation and Solution

In microeconomics, the optimality of Walrasian equilibrium can be achieved by solving the problem of social welfare maximization [29]. Thus, a social welfare maximization problem is proposed in this subsection to search for the Walrasian equilibrium. The designed problem can reconcile the lateral optimization problems of the crowdsourcer and mobile users. Specifically, the solution of the designed problem is supposed to simultaneously solve problem Equations (4) and (6), match the demands with the supplies, and finally reach the Walrasian equilibrium. Thus, the social welfare maximization problem is formulated as:

(9) $$\sum_{k=1}^{M}\left(a_{k}d_{k}^{2}+b_{k}d_{k}\right)-\sigma \prod_{j=1}^{N}D_{j}^{w_{j}},$$

(10) $$\mathrm{subject}\;\mathrm{to}\;D_{j}=\sum_{u_{k}\in A_{j}}d_{k},\;j\in \left\{1,2,\ldots ,N\right\},$$

(11) $${\underline{D}}_{j}\leq D_{j},\;j\in \left\{1,2,\ldots ,N\right\},$$

(12) $$0\leq d_{k}\leq {\bar{d}}_{k},\;k\in \left\{1,2,\ldots ,K\right\}.$$

In Equation (9), instead of maximizing the social welfare J, the minimization of −J is present so that a standard convex optimization problem can be constructed. The constraints in (10) imply a balance between the supply by mobile users and the demand by the crowdsourcer. The constraints in (11) present local boundaries in all subareas for the crowdsourcer, while the constraints (12) serve as local boundaries for all mobile users.

The cost function $a_{k}d_{k}^{2}+b_{k}d_{k}$ of each mobile user $u_{k}$ is convex, while the payoff function $\sigma \prod_{j=1}^{N}D_{j}^{w_{j}}$ of the crowdsourcer is concave. So the optimization problem Equation (9) is a convex problem with affine constraints. Without considering the local constraints (11) and (12), the optimization problem can be transformed into an augmented Lagrangian function as:

(13) $$L=\sum_{k=1}^{M}\left(a_{k}d_{k}^{2}+b_{k}d_{k}\right)-\sigma \prod_{j=1}^{N}D_{j}^{w_{j}}+\sum_{j=1}^{N}\lambda_{j}(D_{j}-\sum_{u_{k}\in A_{j}}d_{k}),$$

where all $\lambda_{j}$ are coupling variables. In Equation (13), all items containing variables $D_{j}$ can be grouped together as well as all items about $d_{k}$. Then Equation (13) is converted to:

(14) $$L=\left(\sum_{j=1}^{N}\lambda_{j}D_{j}-\sigma \prod_{j=1}^{N}D_{j}^{w_{j}}\right)+\sum_{k=1}^{M}[\left(a_{k}d_{k}^{2}+b_{k}d_{k}\right)-\lambda_{j}d_{k}].$$

Subsequently, based on dual decomposition [32], an iterative approach can be developed to solve the original optimization problem. At each iteration, the optimization problem can be formulated as:

(15) $$D_{j}^{r}=arg\;\underset{D_{j}\geq {\underline{D}}_{j}}{min}\overset{N}{\underset{j=1}{\sum }}\lambda_{j}^{r}D_{j}-\sigma \overset{N}{\underset{j=1}{\prod }}D_{j}^{w_{j}},j\in \left\{1,\ldots ,N\right\},$$

(16) $$\forall u_{k}\in U:d_{k}^{r}=arg\underset{0\leq d_{k}\leq {\bar{d}}_{k}}{min}\left(a_{k}d_{k}^{2}+b_{k}d_{k}\right)-\lambda_{j}^{r}d_{k,}$$

(17) $$\lambda_{j}^{r+1}=\lambda_{j}^{r}+\alpha \left(D_{j}^{r}-\underset{u_{k}\in A_{j}}{\sum }d_{k}^{r}\right),j\in \left\{1,\ldots ,N\right\},$$

where $D_{j}^{r}$ and $d_{k}^{r}$ are primal variables at the rth iteration, $\lambda_{j}^{r}$ and $\lambda_{j}^{r+1}$ are dual variables at the *r*th and *r*+1th iteration, respectively, α denotes the searching step size. In fact, the dual variables $\lambda_{j}$ represent the prices $p_{j}$. At each iterative, the original global optimization problem is decomposed into multiple local optimization problems, including *N* local minimization problems corresponding to the subareas and *K* local minimization problems for mobile users. As the results of the *r*th iteration, the demand $D_{j}^{r}$ and the supply $\sum_{u_{k}\in A_{j}}d_{k}^{r}$ are usually not the same. As shown in Equation (17), the mismatch between the demand and the supply updates the prices and drives the procedure to the next iteration. When the mismatch is zero, the iterative process will be converged. Meanwhile all local optimization problems and the original global optimization problem achieve their optimal solution.

As the results of the iterations, the convergent prices, demands and supplies can satisfy the definition of Walrasian equilibrium. It can also be found that the prices of fingerprint collection tasks are determined by both the demands of the crowdsourcer and the supplies of the mobile users. Hence, the market monopoly is eliminated, which is an outstanding advantage of Walrasian equilibrium. Moreover, three-fold optimization is achieved, i.e., the maximization of utilities of the crowdsourcer, mobile users and overall system.

### 4.3. Algorithm Design

In order to realize the aforementioned iterative process, a corresponding algorithm was designed. As described in our system model, the crowdsourcer resides in a centralized cloud platform. This cloud platform gathers information, updates prices, and manages the interaction between the crowdsourcer and mobile users. As shown in Algorithm 1, the cloud first initializes single fingerprint price for each subarea. The initial prices are broadcasted to the crowdsourcer and mobile users, starting the first iteration. According to the initial prices, the crowdsourcer calculates its optimal demands by solving local optimization problems, Equation (15), while the mobile users calculate their supplies by solving problems, Equation (16). Then the crowdsourcer and mobile users report the demands and supplies to the cloud, which updates the prices according to Equation (17). The updated prices are then broadcast to the crowdsourcer and mobile users, starting a new iteration. Through multiple iterations, when the demands match the supplies, the Walrasian equilibrium is achieved, resulting in the optimal fingerprint price vector $p^{*}$ for all subareas, the optimal demand vector $D^{*}$ for all subareas, and the optimal supply vector $d^{*}$ for all mobile users.


**Algorithm 1: Iterative Algorithm to Search for Walrasian equilibrium**
1. The cloud platform announces fingerprint collection tasks, initializes initial prices $p_{0}$ for all subareas, and broadcasts the prices to the crowdsourcer and mobile users.
**Iteration**
2. Under the received prices, the crowdsourcer calculates its local optimal demands by solving local optimization problems Equation (15) and reports the demands to the cloud platform.3. Under the received prices, each mobile user calculates its supplies by solving problems Equation (16) and reports the supplies to the cloud platform.4. Based on the received demands and supplies, the cloud platform first calculates the mismatch between supplies and demands in each subarea, then updates prices, according to Equation (17), and broadcasts the updated prices to the crowdsourcer and mobile users.
**Result**
5. When the mismatch between supply and demands is convergent, results can be obtained, including the optimal price $p^{*}$, the optimal demands $D^{*}$ for the crowdsourcer, and the optimal supplies $d^{*}$ for all mobile users.

## 5. Simulation and Analysis

In this section, the performance of our proposed mechanism was evaluated through extensive numerical simulation. Simulation scenario and parameters were introduced first. Then we verified the convergence and optimality of the proposed algorithm. Finally, we will explain the self-reconfiguration capabilities of the system, indicating the robustness and scalability of the proposed algorithm.

In the simulation, the whole area had four subareas, which were denoted as *A*_1_, *A*_2_, *A*_3_, and *A*_4_. The crowdsourcer hopes to collect fingerprint data from these four sub-areas. Inside *A*_1_, *A*_2_, *A*_3_, and *A*_4_, the number of mobile users were 8, 7, 4, and 3, respectively. All these mobile users were willing to provide fingerprint supply. The cloud set the initial prices for all subareas to 5. Other parameters used in the simulation are listed in Table 1.

### 5.1. Verification of Convergence

**Figure 2:**
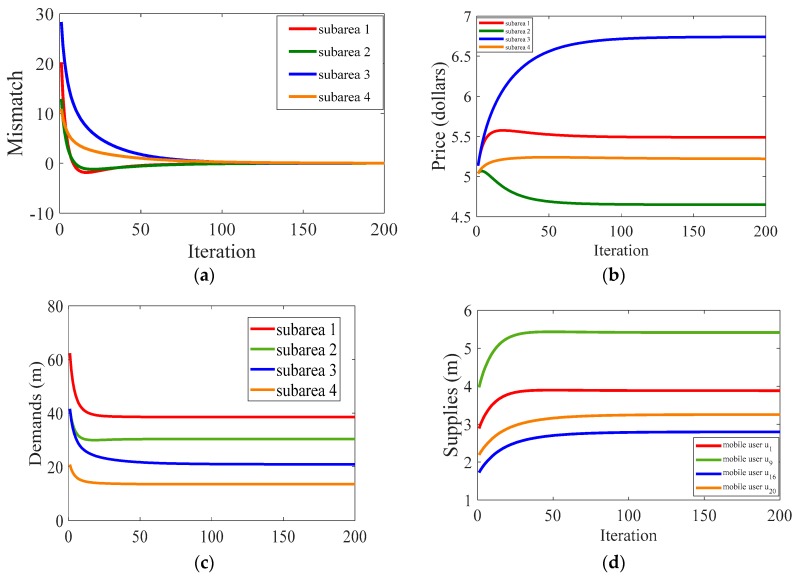
Verification of convergence for (**a**) mismatch between demand and supply, (**b**) price of trajectory, (**c**) demand of the crowdsourcer, and (**d**) supply by mobile user $u_{1},u_{9},u_{16},$ and $u_{20}$.

This subsection verifies the convergence of the proposed algorithm. To this end, Figure 2 shows how the mismatch, the prices, the demands, and the supplies changed with the number of iterations. As seen from Figure 2a, in each subarea, the mismatch between the trajectory requirements of the crowdsourcer and the trajectory supply of mobile users could converge to zero. This convergence means that each subarea has reached the balance between supply and demand, enabling the market to clear. From Figure 2b, it can be observed that the trajectory prices of all subareas tended to be stable along with the iteration progress. In addition, even if the initial prices of these subareas were the same, the convergent prices of all four subareas were different. The reason for this is that the price in one subarea only depends on the pattern of demand and supply in that subarea. In other words, the price is determined by the imbalance between the demand of the crowdsourcer and the supply of mobile users. Neither party alone can monopolize the price, reflecting the fairness of the system. From Figure 2c, it can be observed that the crowdsourcer had different demands in different subareas. The demands all converged to the corresponding optimal values, maximizing the utility of the crowdsourcer under the optimal price. Similarly, as shown in Figure 2d, the supplies of the four mobile users converged to different values. These four mobile users, namely $u_{1},u_{9},u_{16}$ and $u_{20}$, were randomly selected from different subareas. At the convergent point, each mobile user maximized its utility by providing appropriate trajectory supply.

### 5.2. Verification of Optimality

**Figure 3:**
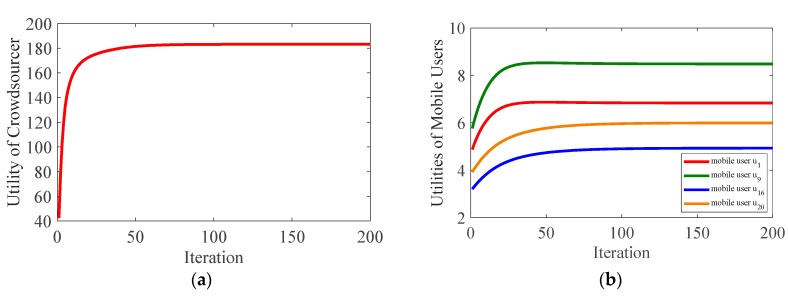

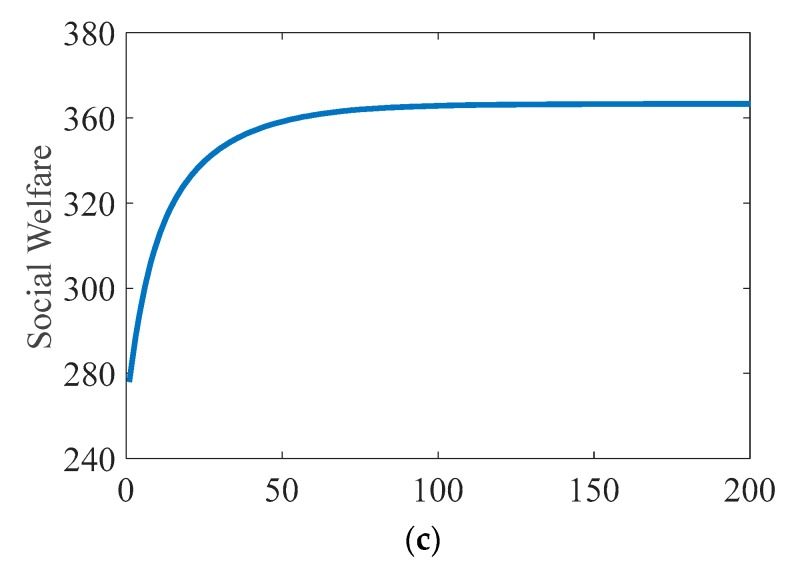
Utility changes with the iteration process in our proposed algorithm: (**a**) utility of the crowdsourcer; (**b**) utility of mobile user $u_{1},u_{9},u_{16}$, and $u_{20}$; and (**c**) social welfare.

**Figure 4:**
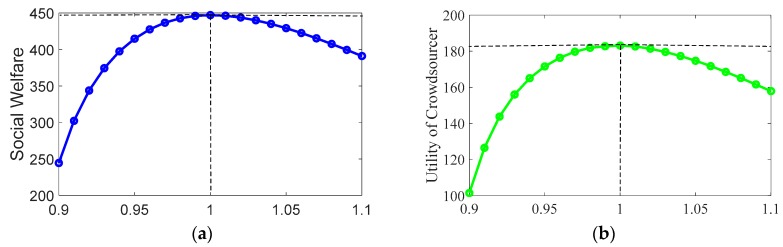

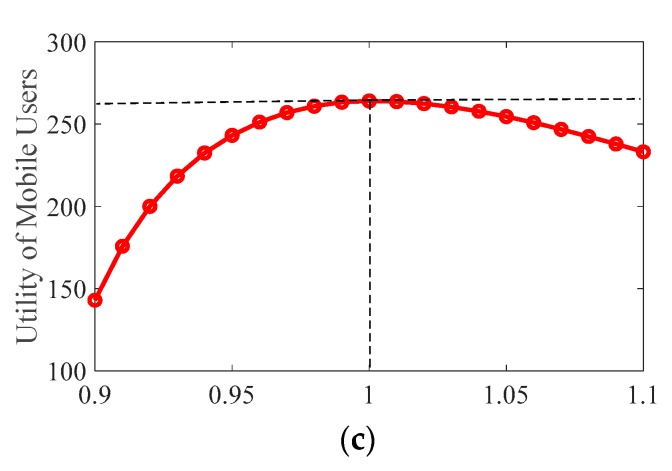
Performance comparison between our proposed algorithm and crowdsourcer-centric scheme: (**a**) social welfare; (**b**) utility of the crowdsourcer; and (**c**) total utility of mobile users.

To verify the optimality of the proposed algorithm, Figure 3 shows how the utility of participators and social welfare changed with the iteration process in our proposed algorithm, while Figure 4 presents the performance comparison between our proposed scheme and crowdsourcer centric scheme [30].

Observed from Figure 3a, the utility of the crowdsourcer increased with the number of iterations and finally achieved its maximum value. In Figure 3b, the utility of mobile users also increased to the optimized values to ensure their profits. In Figure 3c, the social welfare of the whole system grew with the iteration process and finally reached the optimal value, indicating that the social resources were optimally allocated and fully utilized.

In Figure 4, the result of our proposed scheme is represented by the summit, while the curves correspond to the performance of crowdsourcer centric scheme. The basic principle of the latter scheme is explained as follows. The market is assumed to be dominated by the crowdsourcer, which can adjust the obtained stable prices as it wants. For simplicity, the adjusted prices were assumed to range from 90% to 110% of the convergent prices obtained in our proposed scheme. The crowdsourcer adjusts the prices in all subareas simultaneously. Under the adjusted prices, the crowdsourcer calculates the optimal demands in each subarea based on the maximization of its own utility. Then, under the constraint of the match between demand and supply, the supply of each mobile user is calculated based on the maximization of the total utility of all mobile users.

As shown in Figure 4a, social welfare by the crowdsourcer-centric scheme changed with the adjusted prices. We can find that only when the adjusted prices equal the convergent prices in our proposed algorithm, the social welfare reaches its maximum value. As can be seen from Figure 4b, the utility of the crowdsourcer reached the summit only when the adjusted prices are the same as our equilibrium prices. This demonstrates the optimality of our proposed algorithm with regard to the utility of the crowdsourcer. In Figure 4c, it can be observed that when the crowdsourcer manipulates the prices to below the summit prices, the total utility of mobile users are much lower than the maximum value. This phenomenon not only indicates the unfairness of the crowdsourcer centric scheme, but also verifies the optimality of our proposed algorithm in view of the utilities of mobile users.

### 5.3. Self-Reconfiguration Ability

**Figure 5:**
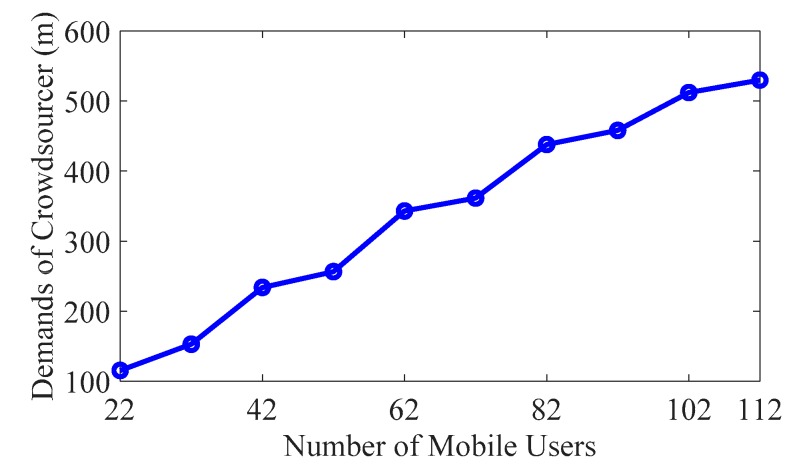
Demands of the crowdsourcer change with the number of mobile users.

In order to illustrate the self-reconfiguration ability of our Walrasian equilibrium-based incentive mechanism, we discuss the impact of available resources, i.e., the number of mobile users performing fingerprint collection tasks. As shown in Figure 5, the total demand of the crowdsourcer increased with the number of mobile users. The total demand is the summation of all demands in four subareas, while the number of mobile users is randomly selected. Along with the number of users, the synchronistic growth of the total demand indicates that the crowdsourcer can adjust its own trajectory requirements according to the change in the number of mobile users. This demonstrates the self-reconfiguration capability of the system. At the same time, this phenomenon also shows that the proposed algorithm has strong robustness and scalability. Therefore, mobile users can easily join or leave the system without affecting the operation of the entire system.

## 6. Conclusions

Mobile crowdsourcing is critical for indoor fingerprinting-based localization systems because it is very helpful for the construction of fingerprint databases. In order to effectively motivate the ubiquitous mobile users to participate in the fingerprint data collection task, this paper proposed a Walrasian equilibrium-based incentive mechanism. First, the utility functions of the crowdsourcer and mobile users were defined, as well as the overall social welfare. Then, in order to reach the Walrasian equilibrium, a social welfare maximization problem was constructed and solved. The dual decomposition method was employed to solve the original optimization problem. The maximization of social welfare was decomposed into the benefit optimization for the crowdsourcer and mobile users. Then, a distributed iterative algorithm was designed to achieve Walrasian equilibrium at which the optimal trajectory data price, trajectory demand, and trajectory supply were determined. Simulation results verified the convergence, the optimality, and the self-reconstruction ability of the proposed incentive scheme.

## Figures and Tables

**Table 1:** Simulation parameters.

Parameter	Value
Crowdsourcer payoff coefficient σ	50
Elastic coefficient $\{w_{1},w_{2},w_{3},w_{4}\}$	{0.3, 0.2, 0.2, 0.1}
Initial prices for all subareas **p**	{5, 5, 5, 5}
Number of users in each subarea $\left\{A_{1},A_{2},A_{3},A_{4}\right\}$	{8,7,4,3}
User cost coefficient $a_{i}$	randomly selected among (0,1)
User cost coefficient $b_{i}$	randomly selected among (0,2)
Iteration coefficient α	default value is 0.005
